# Puerperal Eclampsia

**Published:** 1894-12

**Authors:** L. W. Hollis

**Affiliations:** Abilene, Texas


For Texas Medical Journal.

### PUERPERAL ECLAMPSIA.

BY L. W. HOLLIS, M. D., ABILENE, TEXAS.

Read before Taylor Medical Society August, 1894, and voted to be sent to the Texas Medical Journal.

ECLAMPSIA means convulsions of various kinds, epileptic, hysterical, etc., but there is a special form of convulsion of general uniformity in symptoms and some collateral circumstances, evidently peculiarly connected with pregnancy and parturition, to which the name puerperal convulsion is by general consent given. Its frequency is one in five hundred labors. We find that it is more frequent during labor, but does occur sometimes during pregnancy, and in the puerperal state. It is much more liable to occur in the primapara than multipara.

Regarding the nature and pathogenesis of eclampsia it must be admitted that our knowledge is as yet imperfect, although many theories have been propounded, among them the most important are the following: To lesions of the kidneys which may consist simply of hyperæmia, or if more advanced, of exudation and fatty metamorphosis, or of atropic changes consequent to those previously mentioned.

Allied to this view is that which considers eclampsia a manifestation of uremia or ammonemia, the urea that exists having been oxidized to the further extent of carbonate of ammonia, and in that form contaminating the blood. Some authors lay stress upon conditions of compression as producing renal insufficiency, through interference with the vessels of the kidney. While others believe that it is through encroachment upon the ureters that the kidney becomes affected. I could give many more theories as set forth by eminent authors, but deem it unnecessary. One thing, however, is worth mentioning, and that is, albuminuria in a great majority of cases attends puerperal eclampsia, and in fatal cases well marked lesion of the kidney is found, hence we will say that it is due to that organ. The eclamptic seizure may come on without any warning whatever, though we frequently have some of the following sympoms: Headache, mental depression, dizziness, vertigo, amblyopia, severe gastric pain, with or without nausea, œdema of some part of the body is a very frequent symptom. Though, of course, we don't always have an eclampsia with œdema. Examination of the urine will show albumin, renal epithelium, and hyaline epitheliæ, and granular cast. The attack resembles an epileptic convulsion, without the initial scream. Speaking from a very limited clinical standpoint, I will say that I have noticed in seven cases out of eleven, that warning was given by involuntary twitching of the index finger, and in three cases the patient asked what is the matter with my finger, just as she was seized with the convulsion. The reason that I mention this is to know whether or not others have noticed it. Seeing it in the majority of cases coming under my care, prompted me in calling your attention to it. A slight twitching of the eye lids or the corners of the mouth may precede for an instant the general convulsion, which twists he face to one side, fixes the pupils either in contraction or dilatation, rolls the eye balls, flexes the limbs with tonic and clonic contractions of the muscles, bends the trunk sideways or backward, swells the neck, congests the visage, and consciousness is lost, the mouth foams, the tongue is often injured by biting it, respiration is at first suspended, then becomes stertorous. After a few minutes the twitching ceases, and the patient passes off into the stage of coma. During and preceding the seizure you have a full bounding pulse, and a general rigidity of the muscles, but now the bounding pulse becomes soft, perspiration attends the relaxed state of the muscles and skin, and if no remission follows, she gradually regains her consciousness, and the nerve storm subsides.

The prognosis for mother is not bright, though many cases properly managed recover, while to the child it is exceeding fatal.

As to the treatment, I think that we can do a great deal toward prophylactic treatment by careful attention to the kidneys with diruretics and suitable tonics. The recumbent position is good practice, as it tends to relieve the pressure upon the renal vessels. The diet should be milk. If we have one attack of eclampsia, we may look for repeated attacks until the uterus is emptied, so that the best practice and policy is to hasten labor at once. If dilatation is not complete, it should be done at once manually, and version or forceps used, but of course we can't always deliver just when we wish. We will now consider the best line of treatment during the period of dilatation and eclamptic seizures. In other words, what will we do during the convulsion, etc., until we can deliver the woman? Chloroform given be inhalation during the attack is indispensable, and acts well in cutting it short. If the patient be plethoric, with full and bounding pulse, I say bleed at once and bleed well. This is an old, but I think good treatment, though condemned by some. When the patient is not a subject for bleeding, then we have two chief remedies at hand. One is the hypodermic injection of verat. vir., and the hypodermic use of morphia. I have not had much experience with the verat. vir. treatment, but have used the morphia treatment and found it good. I will now cite you to a case that came under my care recently, giving you the management of it. I was called to a neighboring town on 27th of last month to attend Mrs. S., who formerly was a patron of mine, but then under the care of Dr. McC. I will say here that she had given birth to two children before this, and that I attended her, and in the first labor she had puerperal convulsions with breech presentation of the child. I gave her then chloroform, performed version and delivered her of a still born child. Next labor came on about two years afterward. I had her under my care for three months before confinement, and looked very closely into the action of the kidneys and liver. It came on, natural labor, without any sign of convulsions. This now was her third confinement. She was taken the morning of the 27th at 3 o'clock. The doctor was called. There was no dilatation scarcely, but pains at intervals of fifteen to thirty minntes. She was very nervous during pain, and complained of frontal headache. She remained this way for two hours or more, when she called the attention of the doctor to look at her index finger which was twitching involuntarily, and was seized with a very hard and lasting convulsion. The family had me sent for at once. I reached there in about seven hours afterwards. She had had five convulsions when I arrived. The attending physician had given her chloral internally, and let her inhale chloroform during convulsions; her tongue was badly injured. I made examination (vaginal), and found very little dilatation and the os very high, pains coming about every ten to fifteen minutes and marked nervousness existing, with tendency to convulsions with each pain. My first impulse was to bleed her, for she was a suitable case for it, but decided that I would hold up a while on it, and try morphia hypodermically, knowing that she needed rest, and thinking it would act two-fold by quieting the nervous system and giving rest, thereby assisting dilatation, fully deciding to bleed at once if convulsions returned. I commenced by giving her ½ grain of morphia hypodermically, following with ¼ grain in one hour, giving her ¼ grain three hours later. It acted well, she went into a sound sleep, and remained so for nine hours, without the least nervous symptom. She was awakened by a labor pain. I made examination and found quite a dilated os, with menbrane protruding, pains came on nicely, and I thought it would end without further trouble, but not so; she continued in seemingly natural labor three hours with complete dilatation, but head was not engaged, yet I was expecting it with natural delivery very soon, when she was very suddenly seized with convulsions again. I did not bleed as I expected, but applied forceps at once and delivered her. Dr. McC. give her chloroform during the seizure. The child was seeming dead, yet after some time of constant work at resuscitation it came to life, but not until I had inflated its lungs well with air. The mother had two slight attacks afterwards, but very slight indeed. I gave her potass. bro. with some good diuretics. She made a good recovery. Child also lives and both doing well.

